# Sustainable Management of Filamentous Algae in Freshwater Ecosystems: Insights from *Cladophora* sp. Life History, Reproductive Tactics, and Growth Ecology

**DOI:** 10.3390/biology14121671

**Published:** 2025-11-25

**Authors:** Liangjie Zhao, Liangxin Guo, Chenxi Tan, Yongtao Tang, Yuanye Ma, Zhen Zhang, Yongxu Cheng, Chen Qian

**Affiliations:** 1 School of Fisheries, Xinyang Agriculture and Forestry University, Xinyang 464000, China; a850924t@163.com (L.Z.); tanchenxi2013@163.com (C.T.); t13721071655@126.com (Y.T.); zzhang@xyafu.edu.cn (Z.Z.); 2 Xinyang Fisheries Station, Xinyang 464000, China; xysc6653765@163.com; 3 Xinyang Nanwan Reservoir Affairs Center, Xinyang 464000, China; yuanye19872372@126.com; 4 School of Marine Sciences, Ningbo University, Ningbo 315832, China; chengyongxucrablab@hotmail.com; 5 College of Fisheries and Life Science, Shanghai Ocean University, Shanghai 201306, China

**Keywords:** *Cladophora* sp., life history, reproduction, driven factors, management, freshwater ecosystem

## Abstract

Freshwater ecosystems are increasingly degraded by *Cladophora* blooms, prompting a need to understand its reproductive characteristics for effective control. We combined continuous microscopical monitoring and single-factor and orthogonal experiments, to characterize the life history and reproductive and growth response to different nutrients, pH, temperature, and light conditions of a bloom-forming *Cladophora* sp. A novel mode was observed, in which mature sporangia and gametangia rupture to extrude a pyknotic cytoplasmic mass, inside which zoospores and gametes complete maturation and release. The peak zoospore production occurred in Aquatic No. 6 medium at pH 5, 35 °C, and 54 µmol m^−2^ s^−1^, whereas maximum gamete release was recorded in Knop medium at pH 4, 30 °C, and the same irradiance, with temperature being the principal driver for zoospores and nutrient regime for gametes. Growth trials further revealed that low-N and -P conditions sustain growth, whereas nutrient-rich conditions favor reproduction. In addition, pH 4–5, 35 °C, and 54 µmol m^−2^ s^−1^ conditions favored propagule formation, while pH 7–8, 25 °C, and 81 µmol m^−2^ s^−1^ maximized biomass. These findings provide a basis for predicting *Cladophora* bloom dynamics and offer references for sustainable management of *Cladophora* in freshwater ecosystems.

## Simple Summary

*Cladophora* blooms have become a widespread problem in freshwater ecosystems, degrading water quality and endangering aquatic life. To control its biomass, we systematically examined the life history and reproductive tactics of a freshwater *Cladophora* sp., quantifying propagule release and growth under varying nutrient and ecological conditions. The study uncovered a novel reproductive adaptation: mature zoosporangia or gametangia rupture to extrude a pyknotic cytoplasmic mass, within which zoospores and gametes complete maturation—a key strategy enabling this species to dominate freshwater systems. Additionally, optimal release conditions differ sharply between zoospores and gametes, revealing differentiated responses of zoosporogenesis and gametogenesis to environmental cues. Orthogonal analysis revealed strong synergistic interactions: temperature is the primary driver of zoospore output, whereas nutrient regime governs gamete release. Growth–reproduction trade-offs in *Cladophora* sp. further underpin bloom dynamics: high-N and -P, acidic, and warm conditions favor mass propagule release, whereas neutral–alkaline and moderate-temperature conditions promote biomass accumulation. These findings deliver a predictive framework for forecasting bloom phenology and inform targeted management strategies and highlight the potential of leveraging these physiological responses for controlled cultivation and utilizing *Cladophora* as a biological resource.

## 1. Introduction

Freshwater ecosystems undergoing eutrophication often experience proliferation of filamentous algae, particularly *Cladophora*, which can lead to significant ecological and operational challenges [1,2]. The proliferation of *Cladophora* in freshwater ecosystems is largely driven by its highly efficient reproductive strategies and robust reproductive capabilities. These traits, including both asexual (zoospores) and sexual (gametes) reproductive modes, are pivotal to its life cycle. Distinct life histories characterize different algal species [3], often correlating with their long-term reproductive and growth environments [4]. The algal blooms are not random events but arise from complex interactions between environmental factors and intrinsic species traits [5]. Therefore, a comprehensive understanding of *Cladophora*’s life history is paramount for managing its biomass and predicting its reproductive response to varying environmental conditions.

Temperature and light are critical environmental regulators of algal reproduction and growth [6,7]. Optimal temperatures enhance reproduction [8] and notably shorten reproductive cycles [9]. Temperature is related to the timing of zoospore release of *Cladophora* [10]. Light, acting as the primary energy source, profoundly impacts both the growth and reproduction of algae [11,12]. It is essential for spore germination and early development [13,14]. pH, a key chemistry parameter, influences algal physiological process and photosynthesis [15,16]. Low pH inhibits the photosynthesis of *Chaetomorpha valida* [17]. Furthermore, nutrient availability critically regulates algal growth and reproduction [18]. Nitrogen (N) is a key limiting factor, while phosphorus (P) enrichment (eutrophication) is a primary driver of *Cladophora* overgrowth [19]. Managing N and P inputs is an effective strategy for controlling blooms [20], and the N/P ratio exerts a significant influence on the growth dynamics of *Cladophora* [21]. Moreover, carbon availability also underpins algal growth and metabolism [22].

While the individual effects of temperature, light, pH, and nutrients on algal reproduction are significant, these factors rarely act independently. They frequently interact synergistically [23], and the combined effects of multiple stressors often exert a more pronounced influence on biological processes than individual factors alone [24]. For example, optimal light conditions enhance the utilization efficiency of nitrogen and phosphorus [25], and *Cladophora* in alkaline environments can more efficiently utilize bicarbonate (HCO_3_^−^) for photosynthesis, thereby promoting its growth [26]. Currently, research on the reproductive efficiency of *Cladophora* is limited, with even fewer studies specifically investigating its reproductive responses to individual or, crucially, combined environmental and nutritional factors. Consequently, a comprehensive understanding of how these factors interact and collectively influence *Cladophora* reproduction across diverse environments remains a critical area of ongoing research.

Therefore, this study employed a sequential approach to investigate the reproduction of a freshwater *Cladophora* sp. First, its life history was observed. Subsequently, the effects of four key environmental and nutrient factors on propagule release were investigated to identify optimal reproductive conditions. Building on this, an orthogonal experimental design was utilized to compare the relative strength and interactive effects of different factor combinations on reproduction and finally verify the impact of the factors on growth. The overarching aim is to uncover potential strategies by which nutrient and environmental factors regulate *Cladophora* reproduction and growth, thereby providing crucial references for the healthy and sustainable development of freshwater ecosystems.

## 2. Materials and Methods

### 2.1. Sample Collection and Morphological Observation

Healthy, well-developed filamentous algae were collected from a freshwater pond with a *Cladophora* bloom in Chongming District, Shanghai, China (31°57′84.082″ N, 121°55′32.700″ E). Algal thalli collected from the freshwater pond exhibited bright green coloration, with uniseriate filaments that were sparsely branched (Figure S3). Individual cells were cylindrical, measuring 21–28 µm in diameter and 83–272 µm in length. Chloroplasts were granular and parietal. Morphological and microscopic evaluation confirmed that the samples closely matched the characteristics of *Cladophora* sp.

### 2.2. DNA Extraction, Amplification, and Sequencing

Species identification was performed using combined morphological characterization and 18S rDNA and ITS molecular analysis. After sample collection, genomic DNA was extracted (detailed procedures are shown in the Supplementary Materials). Extracted DNA was temporarily stored at −40 °C and then used for PCR amplification, product purification, and sequencing (Shanghai Majorbio Bio-Pharm Technology Co., Ltd., Shanghai, China). The resulting sequences were compared against the GenBank database. The primers used for eukaryotic 18S rDNA amplification were E4F-17: CTGGTTGATCCTGCCAG and E544R: ACCAGACTTGCCCTCC, and for internal transcribed spacer (ITS) region, they were ITS-9F: 5′-CCGCCCGTCGCTCCTACCGATTGGGTGTG-3′ and ITS-7R: 5′-TCCCTTTTCGCTCGCCGTTACTA-3′ [27,28,29].

The obtained 18S and ITS sequences were submitted to NCBI GenBank and subjected to BLASTn v2.14.0 searches. Reference sequences with >95% coverage and the highest similarity were selected. Published 18S and ITS sequences of *Cladophora* and related genera were retrieved from GenBank. Maximum likelihood phylogenetic trees were constructed separately for 18S and ITS datasets and visualized using FigTree v1.4.4 (Figures S1 and S2). 18S rDNA and ITS sequence identified the isolate as genus *Cladophora*. Although we combined ITS and 18S sequence data with morphological characters, species boundaries within *Cladophora* remain poorly resolved, and neither marker placed the isolate in any recognized species-level lineage. Therefore, to avoid misidentification, we consistently refer to our samples as *Cladophora* sp.

### 2.3. Preparation of Cladophora sp. for Reproduction and Growth Trials

Fresh green filaments were carefully selected and cleaned under a dissecting microscope to remove debris. To eliminate epiphytic contaminants, the filaments were immersed in 0.2% potassium iodide solution for exactly 1 min, followed by four rinses with ultrapure water [30]. The samples were then maintained in ultrapure water under controlled conditions: 25 °C, 60.0 µmol photons m^−2^ s^−1^ irradiance, and a 12 h:12 h light–dark photoperiod [31,32]. During the 48 h acclimation period, the ultrapure water was replaced daily. Uniform and optimal initial culture conditions were applied to all treatments to minimize potential variation and to ensure the validity of the experimental conclusions.

### 2.4. Reproduction Trials

#### 2.4.1. Response of Reproduction to Different Nutrient Conditions and Ecological Factors

The reproduction trials consisted of four single-factor trials with each targeting one influential factor, and two orthogonal experiments focused on zoospore and gamete release, respectively. The response of *Cladophora* sp. to nutrient and ecological conditions was evaluated through four separate single-factor experiments covering culture medium, pH, light, and temperature. Culture media selection was based on five green-algal media in common use: BG11 (Medium A), Aquatic No. 6 (B), Aquatic No. 4 (C), Knop (D), and Chu’s medium (E) [33,34,35,36]. Complete formulations are provided in Table S1. All media were prepared with ultrapure water and analytical-grade reagents (Sinopharm Chemical Reagent Co., Ltd., Shanghai, China). Components were weighed on an analytical balance accurate to 0.0001 g. Three ecological factors, including temperature, light intensity, and pH, were systematically varied to determine their effects on propagule (zoospore and gamete) release. Ranges were chosen according to *Cladophora* tolerance limits (temperature and light) and physiologically relevant extremes (pH) [26,37,38,39,40]. Ecological factor levels were as follows:

- pH: 4, 5, 6, 7, 8, 9, 10, and 11;
- Light intensity: 27.0, 40.5, 54.0, 67.5, 81.0, 94.5, 108.0, and 121.5 µmol photons m^−2^ s^−1^;
- Temperature: 10, 15, 20, 25, 30, 35, and 40 °C.

Each level was set up with three biological replicates. BG11 medium was used throughout all propagation trials, as it yielded the best performance in our preliminary culturing. Light intensity was measured with a photometer, pH was adjusted with HCl/NaOH and recorded with a pH meter, and temperature was monitored with a calibrated thermometer. All experiments were conducted in a programmable incubator (Shanghai Yiheng Technology Instrument Co., Ltd., Shanghai, China) that provides precise control of temperature and illumination.

#### 2.4.2. Orthogonal Experiment Design

Following the single-factor reproductive response investigation, the three best levels of each of the four factors that maximized zoospore release and gamete release were separately identified within the experimental conditions and ecological scope of this study. Two L9 (3^4^) orthogonal arrays, one for zoospore release and one for gamete release, were then constructed to quantify the relative influence of the four factors on propagule release (Table 1). Each combination included three biological replicates, giving fifty-four samples in total for the orthogonal trials. Finally, main effects analysis was performed on the orthogonal results (Tables S1 and S2).

#### 2.4.3. Propagule Observation

Following surface moisture absorption with filter paper, *Cladophora* sp. filaments were aseptically sectioned into 1 cm segments and homogenized. Precisely 0.015 g segments were transferred to 50 mL glass beakers containing 50 mL of culture medium. Segment viability was microscopically assessed at 4 h intervals. After 24 h, 100 µL of homogenized culture suspension was pipetted into a plankton-counting chamber and fixed with Lugol’s iodine solution, and propagules were enumerated. *Cladophora* condition was subsequently monitored. All observations, propagule counts, and imaging employed an Olympus CX33 microscope with an ILAB AL600 camera (Olympus Corporation, Tokyo, Japan). Experiments comprised three independent biological replicates.

### 2.5. Growth Trials

#### 2.5.1. Response of Growth to Different Nutrient Conditions

Nutrient-regulated growth of *Cladophora* sp. was examined in four independent but complementary assays:

- Five formulations: (Culture medium A–E);
- Six nitrogen sources (160 µmol L^−1^ total N): NO_3_^−^-N (N1), NH_4_^+^-N (N2), Urea-N (N3), NO_3_^−^-N:NH_4_^+^-N = 1:1 (N4), NO_3_^−^-N:Urea-N = 1:1 (N5), NH_4_^+^-N:Urea-N = 1:1 (N6), and a N-free control (N0);
- Seven nitrogen concentrations (10 µmol L^−1^ total P): 0, 10, 40, 80, 160, 320, and 500 µmol L^−1^; NO_3_^−^-N was the nitrogen source;
- Eight phosphorus concentrations (160 µmol L^−1^ total N): 0, 1, 3, 6, 10, 15, 25, and 40 µmol L^−1^; K_2_HPO_4_ was the phosphorus source.

All other nutrients were supplied at BG-11 levels. For each treatment, 300 mL of freshly prepared medium was dispensed into 500 mL Erlenmeyer flasks and inoculated with 0.30 g fresh algal mass (surface moisture removed by gentle blotting). Each treatment was replicated six times. During the 8-day incubation, flasks were gently shaken three times daily. Each medium was completely renewed every 48 h, at which point biomass was weighed after blotting. Reagent grades and stock formulations were identical to those detailed in the reproduction trials.

#### 2.5.2. Response of Growth to Different Ecological Factors

To quantify the effects of pH, temperature, and light intensity on the growth of *Cladophora* sp., a three-factor experimental design was established. pH and temperature were adjusted and monitored as described for the reproduction experiment, and light intensity was set at 27.0, 54.0, 81.0, 108.0, and 135.0 µmol photons m^−2^ s^−1^. Sampling and daily culture management regimes were identical to those described for the growth–nutrition experiment. BG-11 medium was used throughout.

#### 2.5.3. Determination of Growth and Antioxidant Parameters

The biomass of *Cladophora* sp. was harvested by gentle vacuum filtration on an 80 μm nylon screen, blotted to a constant wet weight with lint-free paper, and immediately transferred to freshly prepared medium every 48 h. After the 48 h culture period, three randomly chosen replicates per treatment were subsampled (0.10 g fresh weight) and stored at −40 °C pending enzyme assays; thus, these flasks were then removed from the growth evaluation. For antioxidant analyses, frozen tissue was rapidly minced with pre-cooled scissors on ice, weighed, and homogenized in 0.9 mL ice-cold 0.1 M potassium phosphate buffer (pH 7.4, 1:9 *w*/*v*) with an IKA T10 homogenizer (IKA Co., Staufen, Germany) in a 2 mL centrifuge tube. Homogenates were centrifuged at 2500× *g* for 10 min at 4 °C; supernatants were aliquoted, flash-frozen in liquid N_2_, and stored at −40 °C until analysis. Superoxide dismutase (SOD) activity and total antioxidant capacity (T-AOC) were determined with commercial colorimetric kits (Nanjing Jiancheng Bioengineering Institute, Nanjing, China) according to the manufacturer’s protocols for plant tissues.

### 2.6. Statistics Analysis

Statistical analyses were performed using SPSS 19.0 (IBM Corp., Armonk, NY, USA). Data homogeneity was assessed with Levene’s test, with the appropriate transformation (square root, inverse square root, or logarithmic) applied when necessary. The effects of nutrient and environmental conditions on reproductive parameters were analyzed by one-way ANOVA followed by Tukey’s HSD post hoc test. When variance homogeneity remained unattainable after transformation, multiple comparisons were conducted using the Games–Howell test. For orthogonal experimental data, multifactorial ANOVA was employed. Statistical significance was defined as *p* < 0.05.

## 3. Results

### 3.1. Life History Observation

**Figure 1:**
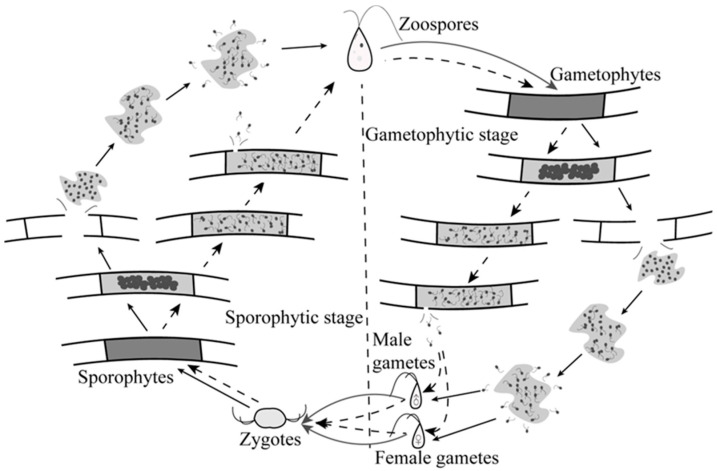
Life cycle schematic of *Cladophora* sp. Solid arrows depict developmental pathways observed in this study; dashed arrows indicate previously reported cycles in the literature.

Unlike the life-history patterns observed in previous discovery studies (Figure 1, dashed arrows), the reproductive process of *Cladophora* sp. was illustrated in a simplified flowchart (Figure 1, solid arrows). Prior to reproduction, cytoplasmic pyknosis formed spherical granules that aggregate into clusters. Mitotic divisions within gametangia produced numerous gamete precursors. Mature zoosporangia or gametangia subsequently ruptured, releasing the pyknotic cytoplasm. Zoospores and gametes then gradually matured within this cytoplasmic mass, developing flagella. Flagellar undulation was observed until propagules were released, a process requiring approximately 30 min and leaving residual grey cytoplasmic debris. Released zoospores (2.0–4.0 µm × 5.0–7.0 µm) exhibited brief swimming motility before settling and germinating over several hours. Gametes (0.5–1.0 µm × 1.0–1.5 µm) displayed vigorous swimming for seconds, with those originating from the same cytoplasmic mass undergoing syngamy. Gamete fusion occurred along the longitudinal axis, forming near-spherical zygotes that subsequently germinated.

### 3.2. Reproductive Response

#### 3.2.1. Nutrient Conditions

**Figure 2:**
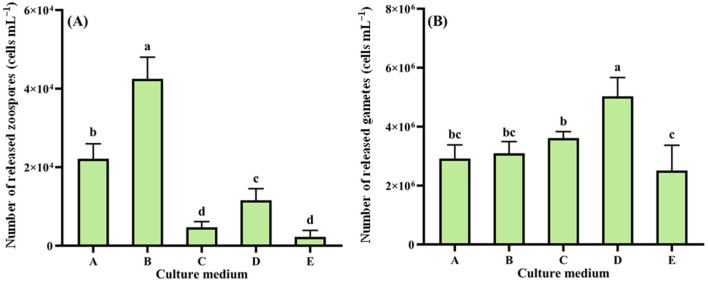
Reproductive response of *Cladophora* sp. to nutritional regimes. (**A**) Zoospores. (**B**) Gametes. Data represent mean ± SD (*n* = 3). Distinct lowercase letters denote statistically significant inter-treatment differences (*p* < 0.05).

The reproductive response of *Cladophora* sp. to nutrient conditions is shown in Figure 2. Zoospore production peaked in Medium B (4.25 × 10^4^ cells mL^−1^, 1.42 × 10^8^ cells g^−1^ wet weight), significantly higher than that in other media (*p* < 0.05, Figure 2A). Minimal production occurred in Medium E (2.26 × 10^3^ cells mL^−1^, 7.54 × 10^6^ cells g^−1^ wet weight), showing no significant difference from Medium C (*p* > 0.05) but significantly lower than Media A, B, and D (*p* < 0.05). For gamete release (Figure 2B), Media D yielded maximum production (5.03 × 10^6^ cells mL^−1^, 1.68 × 10^10^ cells g^−1^ wet weight), significantly surpassing other groups (*p* < 0.05). Media E showed minimal gamete release (2.51 × 10^6^ cells mL^−1^, 8.37 × 10^9^ cells g^−1^ wet weight), significantly lower than Media C and D (*p* < 0.05) but with no significant difference from Media A and B (*p* > 0.05).

#### 3.2.2. pH Levels

**Figure 3:**
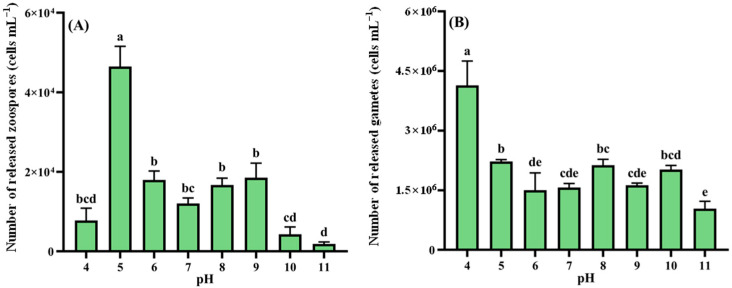
pH-dependent reproductive responses in *Cladophora* sp. (**A**) Zoospores. (**B**) Gametes. Data represent mean ± SD (*n* = 3). Distinct lowercase letters indicate significant inter-pH differences (*p* < 0.05).

Zoospore release across pH gradients is shown in Figure 3A. Maximum production occurred at pH 5 (4.65 × 10^4^ cells mL^−1^, 1.55 × 10^8^ cells g^−1^ wet weight), significantly exceeding all other treatments (*p* < 0.05). The pH 6–9 range showed no significant differences among these four groups (*p* > 0.05), while pH 11 yielded minimal release (1.86 × 10^3^ cells mL^−1^, 6.19 × 10^6^ cells g^−1^ wet weight), significantly lower than all other levels (*p* < 0.05). Gamete release (Figure 3B) peaked at pH 4 (4.14 × 10^6^ cells mL^−1^, 1.38 × 10^10^ cells g^−1^ wet weight), significantly surpassing other pH conditions (*p* < 0.05). Minimal gamete production occurred at pH 11, showing statistical parity with pH 6, 7, and 9 (*p* > 0.05).

#### 3.2.3. Light Intensity

**Figure 4:**
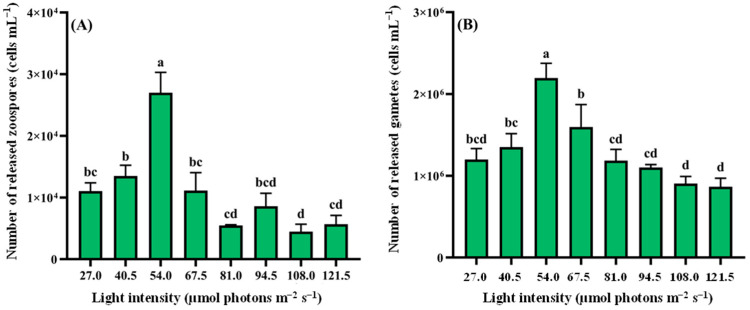
Light-mediated reproductive responses in *Cladophora* sp. (**A**) Zoospores. (**B**) Gametes. Data represent mean ± SD (*n* = 3). Distinct lowercase letters denote significant inter-treatment differences (*p* < 0.05).

Zoospore release showed a unimodal response to increasing light intensity (Figure 4A), peaking at 54.0 µmol photons m^−2^ s^−1^ (2.70 × 10^4^ cells mL^−1^, 9.00 × 10^7^ cells g^−1^ wet weight). This maximum output significantly exceeded all other intensities (*p* < 0.05). Minimal release occurred at 108.0 µmol photons m^−2^ s^−1^, showing significantly lower values than the 27.0–67.5 µmol photons m^−2^ s^−1^ range (*p* < 0.05) but statistical parity with higher intensities (81.0−121.5 µmol photons m^−2^ s^−1^, *p* > 0.05). Gamete release showed a similar pattern (Figure 4B), with optimal production at 54.0 µmol photons m^−2^ s^−1^ (2.20 × 10^6^ cells mL^−1^, 7.33 × 10^9^ cells g^−1^ wet weight), notably higher than other groups (*p* < 0.05). Production declined progressively beyond this intensity, reaching minimum values at 121.5 µmol photons m^−2^ s^−1^ that showed no significant difference from 27.0, 81.0, 94.5, or 108.0 µmol photons m^−2^ s^−1^ groups (*p* > 0.05).

#### 3.2.4. Temperature

**Figure 5:**
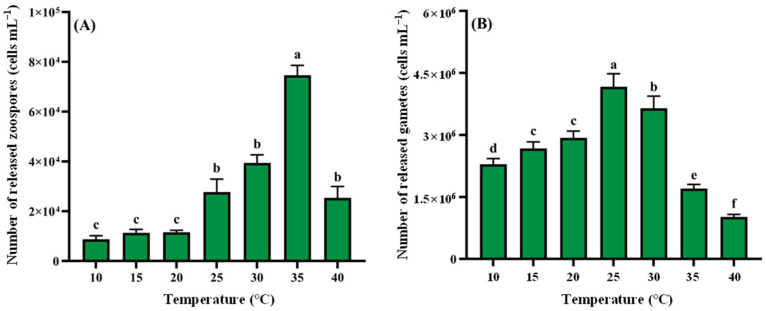
Temperature regulation of reproduction in *Cladophora* sp. (**A**) Zoospores. (**B**) Gametes. Data represent mean ± SD (*n* = 3). Distinct superscript letters denote significant differences among temperature regimes (*p* < 0.05).

The effects of temperature on *Cladophora* sp. reproduction are illustrated in Figure 5. Zoospore release exhibited a unimodal response, with minimum production at 10 °C (8.73 × 10^3^ cells mL^−1^, 2.91 × 10^7^ cells g^−1^ wet weight). Output peaked at 35 °C (7.46 × 10^4^ cells mL^−1^, 2.49 × 10^8^ cells g^−1^ wet weight), significantly exceeding other temperatures (*p* < 0.05). A sharp decline occurred at 40 °C (Figure 5A). Gamete release showed a similar pattern (Figure 5B), increasing at the early stage and decreasing at the late period, maximizing at 25 °C (4.17 × 10^6^ cells mL^−1^, 1.39 × 10^10^ cells g^−1^ wet weight), significantly higher than other treatments (*p* < 0.05). Production decreased progressively to minimum values at 40 °C, significantly lower than other treatments (*p* < 0.05).

#### 3.2.5. Orthogonal Experiment Analysis

**Figure 6:**
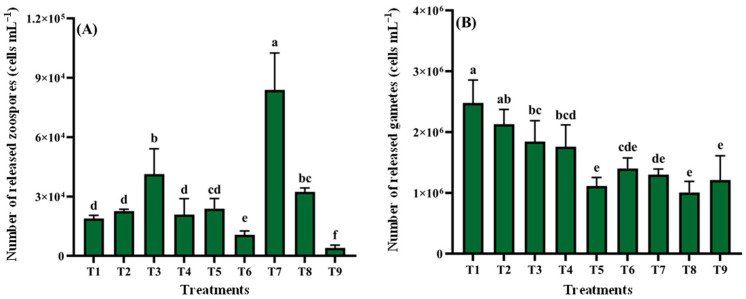
Reproductive responses of *Cladophora* sp. to orthogonal ecological–nutritional matrices. (**A**) Zoospores. (**B**) Gametes. Data represent mean ± SD (*n* = 3). Distinct lowercase letters denote significant differences among treatment groups (*p* < 0.05).

The quantity of zoospore release in all experimental groups is shown in Figure 6. Group T7 yielded maximum zoospore production (8.39 × 10^4^ cells mL^−1^, 2.80 × 10^8^ cells g^−1^ wet weight), significantly higher than that in other groups (*p* < 0.05; Figure 6A). Conversely, T9 showed minimal release (4.03 × 10^3^ cells mL^−1^, 1.34 × 10^7^ cells g^−1^ wet weight), significantly lower than all groups (*p* < 0.05) and <5% of T7 output. Main effect analysis revealed that F-values for nutrient conditions, pH, light intensity, and temperature were 12.113, 10.393, 8.745, and 27.464, respectively (R^2^ = 0.913, Table S2). Gamete release peaked in T1 (2.48 × 10^6^ cells mL^−1^, 8.27 × 10^9^ cells g^−1^ wet weight), while T8 exhibited minimal values (1.01 × 10^6^ cells mL^−1^, 3.37 × 10^9^ cells g^−1^ wet weight), without significant differences compared to groups T5, T6, T7, and T9. Orthogonal analysis indicated that F-values for nutrient conditions, pH, light intensity, and temperature were 30.039, 6.208, 0.191, and 2.471, respectively (R^2^ = 0.812; Table S3).

### 3.3. Growth Response

#### 3.3.1. Growth Response to Nutrient Conditions

**Figure 7:**
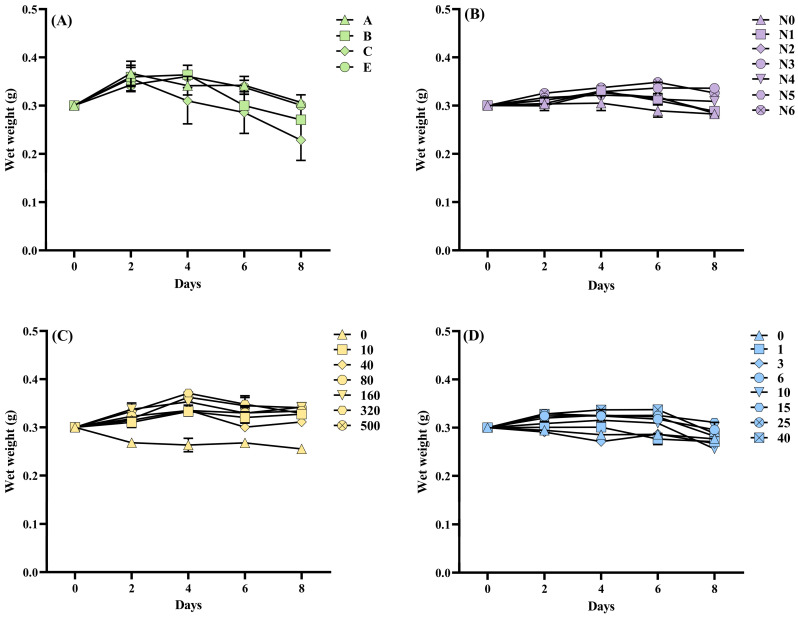
Changes in wet weight in *Cladophora* sp. in response to nutritional regimes. (**A**) Culture media. (**B**) N sources. (**C**) N concentrations. (**D**) P concentrations. Data represent mean ± SD (*n* = 3).

**Figure 8:**
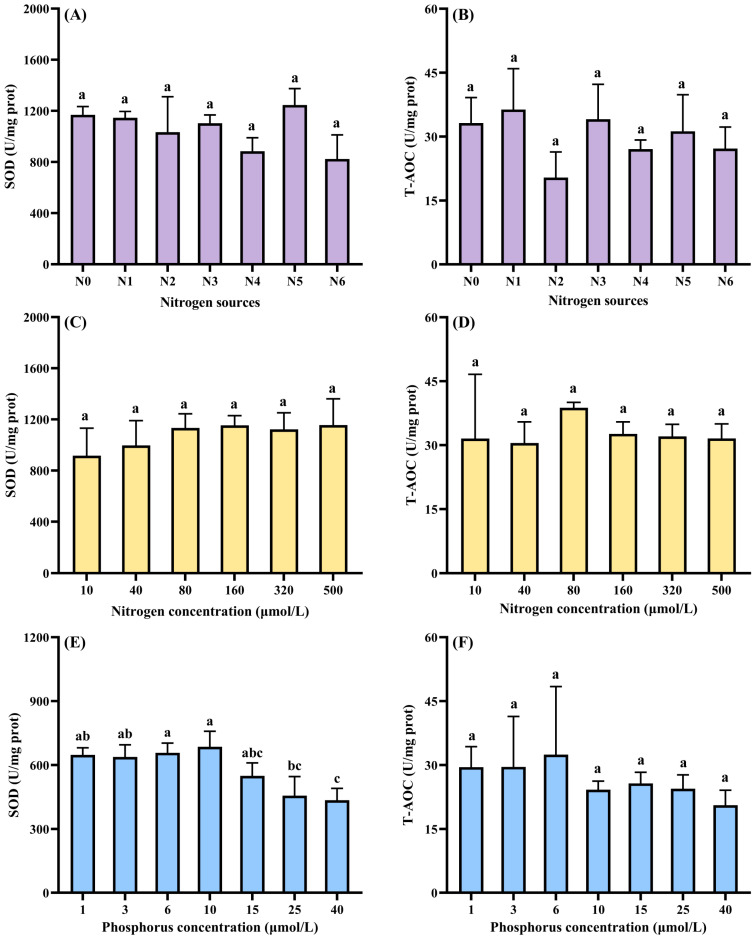
Antioxidant responses of *Cladophora* sp. to nutritional regimes. (**A**) SOD of N sources; (**B**) T-AOC of N sources; (**C**) SOD of N concentrations (**D**) T-AOC of N concentrations; (**E**) SOD of P concentrations; (**F**) T-AOC of P concentrations. Data represent mean ± SD (*n* = 3). Different lowercase letters in the bars denote significant differences among treatment groups (*p* < 0.05).

Nutrient-dependent growth trajectories of *Cladophora* sp. are summarized in Figure 7. Medium D was excluded because bleaching and decomposition occurred during the trial period. Irrespective of treatment, wet weight increased on day 2, and the largest increment was recorded in Medium A (Figure 7A). Thereafter, wet weight in Media B and E continued to rise modestly until day 4, whereas Media A and C entered a gentle decline. All treatments lost biomass rapidly on day 6. However, it was found that the bottom and side walls of the flasks in groups A, B, C, and E were attached with a large number of newly germinated seedlings, especially in groups A and C (Figure S4). Differential nitrogen sources produced distinct growth performance (Figure 7B). The N0 group consistently yielded the lowest wet weight, whereas N6 sustained the highest under the same culture period. The N1–N5 groups followed a parallel pattern, peaking on day 4 and declining thereafter, while the N3 and N6 groups grew continuously until day 6. However, there were no significant differences in SOD or T-AOC among N source treatments (*p* > 0.05; Figure 8A,B). N concentration exerted a pronounced dose-dependent effect (Figure 7C). *Cladophora* sp. cultured in the N-free control lost wet weight continuously, whereas all other N concentration groups gained weight until day 4 and declined subsequently. Higher N concentrations consistently supported greater wet weight at the same culture duration, yet neither SOD nor T-AOC activities differed among N levels (*p* > 0.05; Figure 8C,D). P availability became growth-limiting factor below 3 µmol L^−1^; at these concentrations, wet weight remained at or below the initial weight for the entire culture period (Figure 7D). Once P exceeded 10 µmol L^−1^, wet weight increased until day 4, plateaued or declined modestly on day 6, and decreased sharply thereafter. Among the P concentrations, 40 µmol L^−1^ produced the highest wet weight during the first 6 days. With the increase in P concentrations, the SOD activity first increased and then decreased. SOD values in the 25 and 40 µmol L^−1^ groups were significantly lower than the 6 and 10 µmol L^−1^ treatments (*p* < 0.05). However, there was no significant difference in T-AOC among the groups (*p* > 0.05; Figure 8E,F).

#### 3.3.2. Growth Response to Ecological Factors

**Figure 9:**
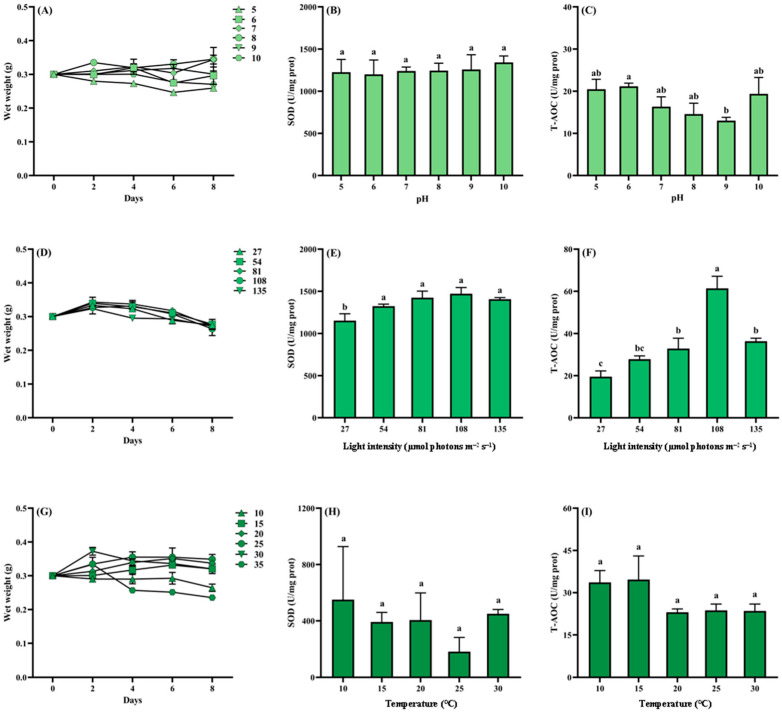
Growth and antioxidant responses of *Cladophora* sp. to ecological factors. (**A**) Wet weight of pH levels; (**B**) SOD of pH levels; (**C**) T-AOC of pH levels; (**D**) wet weight of light intensity levels; (**E**) SOD of light intensity levels; (**F**) T-AOC of light intensity levels; (**G**) wet weight of temperature levels; (**H**) SOD of temperature levels; (**I**) T-AOC of temperature levels. Data represent mean ± SD (*n* = 3). Different lowercase letters in the bars denote significant differences among treatment groups (*p* < 0.05).

Mortality and decay phenomena observed under extreme pH conditions (pH 4 and 11) and at a temperature of 40 °C were excluded from the analysis. As illustrated in Figure 9A, under pH 5, 6, and 10, the wet weight of *Cladophora* sp. declined over time, with the most pronounced reduction at pH 5, followed by pH 10. In contrast, *Cladophora* maintained growth throughout the cultivation period at pH 7 and 8. No significant differences in SOD activity were observed across pH treatments ranging from 5 to 10 (*p* > 0.05). T-AOC exhibited a trend of initial decline followed by recovery with increasing pH, reaching its lowest point at pH 9. Notably, the T-AOC at pH 9 was significantly lower than that at pH 6 (*p* < 0.05; Figure 9B,C). As shown in Figure 9D, under varying light intensities, the wet weight of *Cladophora* sp. followed a consistent trend, an initial increase on day 2, followed by a gradual decline. However, the rate of decline differed among treatments. The highest wet weight was observed under 81 µmol photons m^−2^ s^−1^. SOD activity in *Cladophora* sp. increased progressively with light intensity, with significantly lower activity observed at 27 µmol photons m^−2^ s^−1^ compared to other treatments (*p* < 0.05), though no significant differences were detected among the remaining groups (*p* > 0.05). T-AOC levels initially increased and then decreased with rising light intensity, peaking in the 108 µmol photons m^−2^ s^−1^ group, which was significantly higher than all other groups (*p* < 0.05), and the lowest T-AOC was recorded in the 27 µmol photons m^−2^ s^−1^ group (Figure 9E,F). As depicted in Figure 9G, the wet weight of *Cladophora* sp. increased most rapidly at 25 °C and declined most rapidly at 35 °C. On day 2, all treatments except the 10 °C group exhibited an increase in wet weight, with the greatest increase observed at 30 °C. As shown in Figure 9H,I, no significant differences in SOD activity or T-AOC were detected among temperature treatments (*p* > 0.05).

## 4. Discussion

### 4.1. Life History

Due to high morphological variability, species identification within *Cladophora* is challenging. The ITS, including ITS1 and ITS2, evolves relatively rapidly and exhibits high interspecific polymorphism, making it suitable for low-level taxonomic analysis. Marks and Cummings [41] first used ITS to identify freshwater *Cladophora* from different habitats. Therefore, we combined morphological identification with ITS sequencing for molecular confirmation. Our sample’s ITS region showed >98% similarity to *Cladophora* sp. z-2016. Combined with morphological observations, this confirms that our sample belongs to a species within the genus *Cladophora*. We acknowledge this as an inherent limitation of the present study and recommend that future work incorporate additional markers and culture experiments to achieve species-level resolution.

*Cladophora* demonstrates remarkable adaptability, rapid growth, and robust reproductive capability across diverse environments—traits attributable to its life-history strategy [42]. Investigating dominant *Cladophora* sp. in ponds elucidates bloom formation mechanisms in freshwater ecosystems, providing critical insights for biomass management. This study verified and extended the current understanding of *Cladophora* life history. While prior studies documented isomorphic alternation of generations [43], the *Cladophora* studied here exhibited a novel reproductive mode. Unlike typical *Cladophora* species, gametangia and zoosporangia lack lateral apertures for propagule release. Instead, these structures fracture to expel pyknotic cytoplasm, within which propagules gradually mature. This strategy enables efficient mass discharge from confined spaces, enhancing gamete fusion probability. Such reproductive specialization may represent habitat adaptation. Parodi and Cáceres [44] reported unique autogamy in *Cladophora surera*—unobserved in other congeners. Similarly, the fracture-based mechanism identified here distinguishes this *Cladophora* species and likely underpins its dominance in freshwater ecosystems.

### 4.2. Reproductive and Growth Response to Nutrient Conditions

Filamentous algal blooms in freshwater systems are primarily driven by anthropogenic nutrient loading [45]. N and P are regarded as the key limiting factors for algae growth. Before initiating algal assays, selecting an appropriate culture medium is essential. Given that *Cladophora* belongs to the phylum Chlorophyta, five commonly used media for this group were employed in this study. Our experimental design thus eliminated nutrient limitation for reproduction. Significant differences in zoospore and gamete production among media demonstrated that propagule release is selectively influenced by nutrient composition, ionic profile, and solution chemistry. Zoospore release peaked in Medium B, in which urea was the sole nitrogen source, indicating that urea-N favors zoospore formation in *Cladophora* sp. and highlighting the central role of N source in algal proliferation [46]. Zoospore yield in Medium A (high NO_3_^−^) was second only to that in Medium B and significantly exceeded the remaining three treatments, confirming that elevated nitrate also promotes reproduction. Gamete production was highest in Medium D, which contained the greatest H_2_PO_4_^−^ concentration, consistent with the findings of Malkin et al. [47]. Nevertheless, gametes were still released in Medium A despite its extremely low P content, underscoring the exceptional P sensitivity of *Cladophora* sp. [48,49]. These complementary nutrient strategies underpin *Cladophora* sp. dominance in freshwater systems.

As the culture progressed, the wet weights of *Cladophora* sp. did not increase rapidly under nutrient-replete conditions but declined instead. The attachment of newly germinated seedlings observed indicated that when nutrients are abundant and other conditions are favorable, the alga shifts resources to reproduction and releases large numbers of propagules, reducing biomass. This response is consistent with the species’ life-history strategy. Among the N sources tested, the N6 group (NH_4_^+^: urea-N = 1:1) produced the highest wet weight gain, outperforming the N3 treatment (urea only). Thus, urea alone is a less readily utilized N source for *Cladophora* sp. than NH_4_^+^, corroborating Ross et al. [50]. Remarkably, *Cladophora* sp. in the N6 group exceeded the N2 treatment (NH_4_^+^-only) at equivalent total N, suggesting that in an environment with a low available concentration of nitrogen, the growth of *Cladophora* sp. is prioritized, reflecting the ability to adapt to low nutrient concentrations [51]. However, in an environment with a high concentration of nitrogen, *Cladophora* sp. tends to reproduce, which is consistent with the previous reproductive response results. Over the gradient of 10–500 µmol L^−1^ total N, biomass rose to a maximum at 320 µmol L^−1^ and declined, similar to the results of Zeng [52] in *Cladophora expansa*. Neither SOD activity nor T-AOC differed significantly across the N gradient, demonstrating that the *Cladophora* sp. used in this experiment has a strong tolerance to low- or high-nitrogen environments. Previous studies have shown that the growth of *Cladophora* is limited by P concentrations [49]. In this experiment, KH_2_PO_4_, which performed well in the reproduction section, was selected as the P source for the growth trial. No wet weight gain was observed when the P concentration was <3 µmol L^−1^, whereas maximum biomass was recorded at 40 µmol L^−1^, confirming that increasing the P concentration could promote the growth of *Cladophora* sp. SOD activity declined with increasing P concentrations, dropping sharply at 10 µmol L^−1^, a threshold that apparently relieves P starvation stress.

### 4.3. Reproductive and Growth Response to Ecological Factors

Algal distribution correlates strongly with pH-mediated carbon uptake [53,54]. Although *Cladophora* typically thrives at pH > 7 [55] and zoospore release peaks at pH 7.9–8.3 [10], we observed maximal propagule release at pH 4–5. Although pH 3 might further elevate reproductive output, it exceeds the tolerance threshold of the present strain and was therefore not pursued. Two mechanisms may explain this paradox. (i) Acidic stress suppresses vegetative growth (photosynthetic inhibition, slowed cell division) and disrupts Ca^2+^ homeostasis in the cell wall [56,57]. The resulting physiological imbalance triggers signaling cascades that reallocate resources toward reproduction, accelerating propagule formation and release [58]. (ii) Low pH up-regulates cellulases while inhibiting protease activity [59,60], increasing wall porosity and reducing mechanical strength; localized wall softening thereafter facilitates propagule maturation and release [61]. The specific acid-tolerance mechanisms await future transcriptomic or proteomic validation. Accordingly, during the growth trial, the wet weight of *Cladophora* sp. decreased more rapidly at pH 5 and 6. However, a pH range of 7 to 8 was more conducive to the increase in wet weight. T-AOC reflects the overall antioxidant capacity of cells, encompassing enzymatic and non-enzymatic antioxidants. In this study, the T-AOC level in the pH 5 group was significantly higher than that in the pH 9 group. This might be attributed to the accumulation of non-enzymatic antioxidants such as α-tocopherol during the reproduction process of *Cladophora* sp. [62], consistent with findings that low pH enhances algal reproductive output [63].

Light is crucial for algal growth and development [64]. It has been reported that *Cladophora glomerata* had higher germination and growth rates of akinetes at 50.0 µmol photons m^−2^ s^−1^ [65]. Our identified optimum is 54.0 µmol photons m^−2^ s^−1^, which also explains why the wet weight of *Cladophora* sp. decreased more significantly at 54 µmol photons m^−2^ s^−1^ than at 27 µmol photons m^−2^ s^−1^. *C. glomerata* grows faster under high-light conditions than under low-light conditions [42], and in our experiment, the maximum wet weight occurred at 81 µmol photons m^−2^ s^−1^. Additionally, both SOD and T-AOC activities increased initially with light intensity and then decreased. T-AOC was significantly highest at 108 µmol photons m^−2^ s^−1^, indicating that the light saturation point was close to this value. Intensities exceeding this threshold might have caused chlorophyll degradation or antioxidant system damage, leading to reduced T-AOC [66].

Temperature critically regulates reproductive geography [67]. Zoospore production peaked at 35 °C. This highlights the potential misinterpretation of summer die-offs as mortality rather than recruitment events. Robinson and Hawkes [68] reported that *C. glomerata* grew optimally at around 20 °C, with growth rates declining significantly as temperatures rose from 25 °C to 30 °C. The growth response of *Cladophora* sp. was consistent with these findings. The most pronounced decrease in wet weight occurred at 35 °C, coinciding with massive zoospore release. This reproductive strategy under high temperatures reflects environmental adaptability, as also observed in *Rhizoclonium riparium* [69]. Such plasticity underscores asexual reproduction as a key adaptive strategy for *Cladophora* sp. under climate warming, particularly in shallow freshwater ecosystems vulnerable to temperature fluctuations.

### 4.4. The Synergy of Driving Factors and Implications for Management

Algal biomass seasonality reflects temperature and light fluctuations [70], and *Cladophora* biomass is influenced by nutrients, depth, temperature, and benthic biomass [71]. Our orthogonal experiment identified optimal combinations: condition T7 (Medium C, pH 5, 35 °C, 54 µmol photons m^−2^ s^−1^) promotes zoospore release, whereas T1 (Medium A, pH 4, 20 °C, 67.5 µmol photons m^−2^ s^−1^) favors gamete release. The differences in the optimal conditions for zoosporogenesis and gametogenesis exhibit complementary temperature- and irradiance-dependent responses that align with the documented photosynthetic plasticity of *C. glomerata* [72], indicating an environmentally driven shift in reproductive strategy. In fact, during the transition from spring to summer, the temperature and light intensity are within the suitable range for *Cladophora* sp., thereby stimulating the propagule release. The aforementioned elevated N and P concentrations promote the reproductive success of *Cladophora* sp. Climate change may exacerbate eutrophication [73], and nutrient surges from uneaten feed caused by rising temperatures further amplify reproductive efficiency, leading to the outbreak of *Cladophora* sp. in late spring and early summer. Currently, no universally cost-effective control measures exist. In aquaculture production, preventive actions in winter are recommended: sediment removal, pond draining, sun-drying to reduce nutrient loads, and/or quicklime application to raise pH and inhibit propagule production. In spring, interventions can include applying biological or fermented fertilizers to promote beneficial algae and/or increasing water depth to reduce light penetration and bottom irradiance. Summer vigilance should focus on monitoring reproduction during high-temperature periods. For industrial cultivation, our findings enable targeted biomass regulation strategies optimized for cellulose and carbohydrate extraction. These approaches facilitate sustainable wastewater treatment applications [74,75], providing a cost-effective alternative to traditional treatment methodologies [76]. This dual-purpose valorization transforms *Cladophora* from an ecological challenge into a valuable bioresource. This study acknowledges laboratory constraints, including simplified environmental simulations and limited temporal scales. Future work should integrate field monitoring with predictive modeling to advance understanding of *Cladophora*’s ecological responses across spatial and temporal scales.

## 5. Conclusions

This study provides the first report of a unique reproductive strategy in freshwater *Cladophora* sp., in which propagules complete maturation within an extruded, pyknotic cytoplasmic mass—an adaptation likely central to the alga’s rapid expansion. Systematic evaluation of propagule release across nutrient regimes and ecological gradients, coupled with orthogonal experiments, revealed that zoospore and gamete production are governed by distinct optima of nutrients, pH, light intensity, and temperature. Temperature predominantly controls zoospore output, whereas nutritional conditions are the primary driver of gamete release. Integration with growth assays further demonstrated a trade-off strategy: reproduction is prioritized under nutrient-replete (N ≥ 40 µmol L^−1^, P ≥ 6 µmol L^−1^) conditions, while growth dominates under nutrient-deficient (N ≤ 10 µmol L^−1^, P ≤ 3 µmol L^−1^) conditions in this study. A low pH of 4–5 and a high temperature of 35 °C stimulate propagule formation but suppresses growth. These findings offer new insight into the ecological adaptability and bloom mechanisms of *Cladophora* and highlight differential reproductive ecology as a foundation for targeted management strategies supporting the sustainable development of freshwater ecosystems.

## Figures and Tables

**Table 1:** Orthogonal experimental design.

Treatments	Zoospore				Gamete			
	Culture Medium	pH	Temperature (°C)	Light Intensity (μmol·m^−2^·s^−1^)	Culture Medium	pH	Temperature (°C)	Light Intensity (μmol·m^−2^·s^−1^)
T1	A	5	25	40.5	C	4	20	67.5
T2	A	6	30	54.0	C	8	25	54.0
T3	A	9	35	67.5	C	10	30	40.5
T4	B	5	30	67.5	D	4	25	40.5
T5	B	6	35	40.5	D	8	30	67.5
T6	B	9	25	54.0	D	10	20	54.0
T7	D	5	35	54.0	B	4	30	54.0
T8	D	6	25	67.5	B	8	20	40.5
T9	D	9	30	40.5	B	10	25	67.5

## Data Availability

The original data presented in the study are openly available in Zenodo at [https://doi.org/10.5281/zenodo.17452747].

## Supplementary Materials

The following supporting information can be downloaded at: https://www.mdpi.com/article/10.3390/biology14121671/s1, Genomic DNA extraction protocol; Table S1: The detailed recipes for the culture media used in the experiment; Table S2: ANOVA of number of released zoospores as influenced by culture medium, pH, temperature, and light intensity; Table S3: ANOVA of number of released gametes as influenced by culture medium, pH, temperature, and light intensity; Figure S1: Phylogenetic tree of *Cladophora* sp. based on 18S rDNA gene sequences; Figure S2: Phylogenetic tree of *Cladophora* sp. based on ITS sequences; Figure S3: Photos of branching conditions of the *Cladophora* sp. used in this study; Figure S4: The newly germinated seedlings of *Cladophora* sp. attached the bottom and side walls of the flasks observed during the growth trials.

## Abbreviations

The following abbreviations are used in this manuscript:

N	Nitrogen
P	Phosphorus
SOD	Superoxide dismutase
T-AOC	Total antioxidant capacity
HCO_3_^−^	Bicarbonate
ITS	Internal transcribed spacer
