# Supplementary material for: Sustainable Management of Filamentous Algae in Freshwater Ecosystems: Insights from Cladophora sp. Life History, Reproductive Tactics, and Growth Ecology
Source: Biology (Basel). 2025 Nov 25;14(12):1671. doi: 10.3390/biology14121671 (PMC12729994; doi:10.3390/biology14121671)
Supplement: Supplementary file 1 [file biology-14-01671-s001.zip › biology-3976815-supplementary.pdf]

Genomic DNA extraction protocol

① Weigh 200 mg of fresh green filaments samples. Cut into small pieces and place in a mortar. Add liquid nitrogen to fully freeze the tissue, then quickly and vigorously grind into a fine powder. Add liquid nitrogen intermittently during grinding to prevent thawing. After thorough grinding, place the mortar in a 56°C water bath until the sample powder just begins to thaw.
② Add 350 µl PBS and 0.9 µl RNase A stock solution, grind vigorously for 30 seconds, then transfer 350 µl of the ground homogenate to a 2 ml centrifuge tube. If the homogenate volume is less than 350 µl, supplement with PBS to 350 µl.
③ Add 150 µl Buffer C-L and 20 µl Proteinase K. Vortex immediately for 1 min to mix thoroughly. After brief centrifugation, incubate the tube in a 56°C water bath for 10 min.
④ Add 350 µl Buffer P-D, vortex for 3 s to mix thoroughly, then centrifuge at 12,000 g for 10 min.
⑤ Place the DNA preparation column into a 2 ml centrifuge tube. Transfer the mixture from step 4 onto the column, then centrifuge at 12,000 g for 1 min.
⑥ Discard the flow-through, place the column back into the original 2 ml tube, add 500 µl Buffer W1, and centrifuge at 12,000 g for 1 min.
⑦ Discard the flow-through, place the column back into the original 2 ml tube, add 700 µl Buffer W2, and centrifuge at 12,000 g for 1 min. Repeat this wash step once more with 700 µl Buffer W2. Then discard the flow-through and centrifuge again at 12,000 g for 1 min to remove residual buffer.
⑧ Transfer the DNA preparation column to a clean 1.5 ml centrifuge tube. Add 100–200 µl Eluent or deionized water to the centre of the membrane, let stand at room temperature for 1 min, then centrifuge at 12,000 g for 1 min to elute the DNA.

Supplementary Table S1. The detailed recipes for the culture media used in the experiment.

Component	Culture Medium				
	A	B	C	D	E
$Ca(NO_3)_2$	-	-	-	1.000	0.040
$(NH_4)_2SO_4$	-	-	0.200	-	-
$CaCl_2 \cdot 2H_2O$	0.036	-	-	-	-
KH_2PO_4	-	-	-	0.250	-
$Ca(H_2PO_4)_2$	-	-	0.030	-	0.100
$MgSO_4$	-	-	0.080	-	0.025
KCl	-	0.033	0.025	0.125	-
$CaCl_2$	-	0.030	-	-	-
$NaHCO_3$	-	0.100	0.100	-	-
Na_2CO_3	0.020	-	-	-	0.020
$FeCl_3$, 1% aqueous solution	-	-	1d	1d	1d
Na_2SiO_3	-	-	-	-	0.025
Soil leach solution	-	0.500	0.500	-	-
K_2HPO_4	0.040	-	0.010	-	0.010
Citric acid	0.006	-	-	-	-
$FeSO_4$, 1% aqueous solution	-	0.200	-	-	-
$NaNO_3$	1.500	-	-	-	-
CH_4N_2O	-	0.133	-	-	-
H_3PO_4	-	0.033	-	-	-
$MnSO_4 \cdot 7H_2O$	0.075	0.100	-	-	-
Ferric ammonium citrate	0.006	-	-	-	-
$EDTANa_2$	0.001	-	-	-	-
A5	1.000	-	-	-	-

Note: A5 includes H_3BO_3 (2.86 $g \cdot L^{-1}$), $MnCl_2 \cdot 4H_2O$ (1.86 $g \cdot L^{-1}$), $ZnSO_4 \cdot 7H_2O$ (0.22 $g \cdot L^{-1}$), $Na_2MoO_4 \cdot 2H_2O$ (0.39 $g \cdot L^{-1}$), $CuSO_4 \cdot 5H_2O$ (2.86 $g \cdot L^{-1}$) and $Co(NO_3)_2 \cdot 6H_2O$ (0.05 $g \cdot L^{-1}$). Culture media "BG11", "Aquatic No. 6", "Aquatic No. 4", "Knop" and "Chu's" were marked as Medium A, B, C, D and E, respectively.

Supplementary Table S2. ANOVA of number of released zoospores as influenced by culture medium, pH, temperature and light intensity.

Source	Type III Sum of Squares	df	Mean Square	F	Sig.
Corrected Model	1.274E10	8	1.593E9	18.308	.000
Intercept	1.576E10	1	1.576E10	181.198	.000
Culture medium	2.108E9	2	1.054E9	12.113	.001
pH	1.808E9	2	9.042E8	10.393	.002
Temperature	4.779E9	2	2.389E9	27.464	.000
Light intensity	1.522E9	2	7.609E8	8.745	.003
Error	1.218E9	14	8.700E7		
Total	3.499E10	23			
Corrected Total	1.396E10	22			

a. R Squared=.913(Adjusted R Squared=.863)

Supplementary Table S3. ANOVA of number of released gametes as influenced by culture medium, pH, temperature and light intensity.

Source	Type III Sum of Squares	df	Mean Square	F	Sig.
Corrected Model	6.017E12	8	7.521E11	9.727	.000
Intercept	6.764E13	1	6.764E13	874.792	.000
Culture medium	4.645E12	2	2.322E12	30.039	.000
pH	9.600E11	2	4.800E11	6.208	.009
Temperature	3.821E11	2	1.910E11	2.471	.113
Light intensity	2.955E10	2	1.477E10	.191	.828
Error	1.392E12	18	7.732E10		
Total	7.504E13	27			
Corrected Total	7.408E12	26			

a. R Squared=.812(Adjusted R Squared=.729)

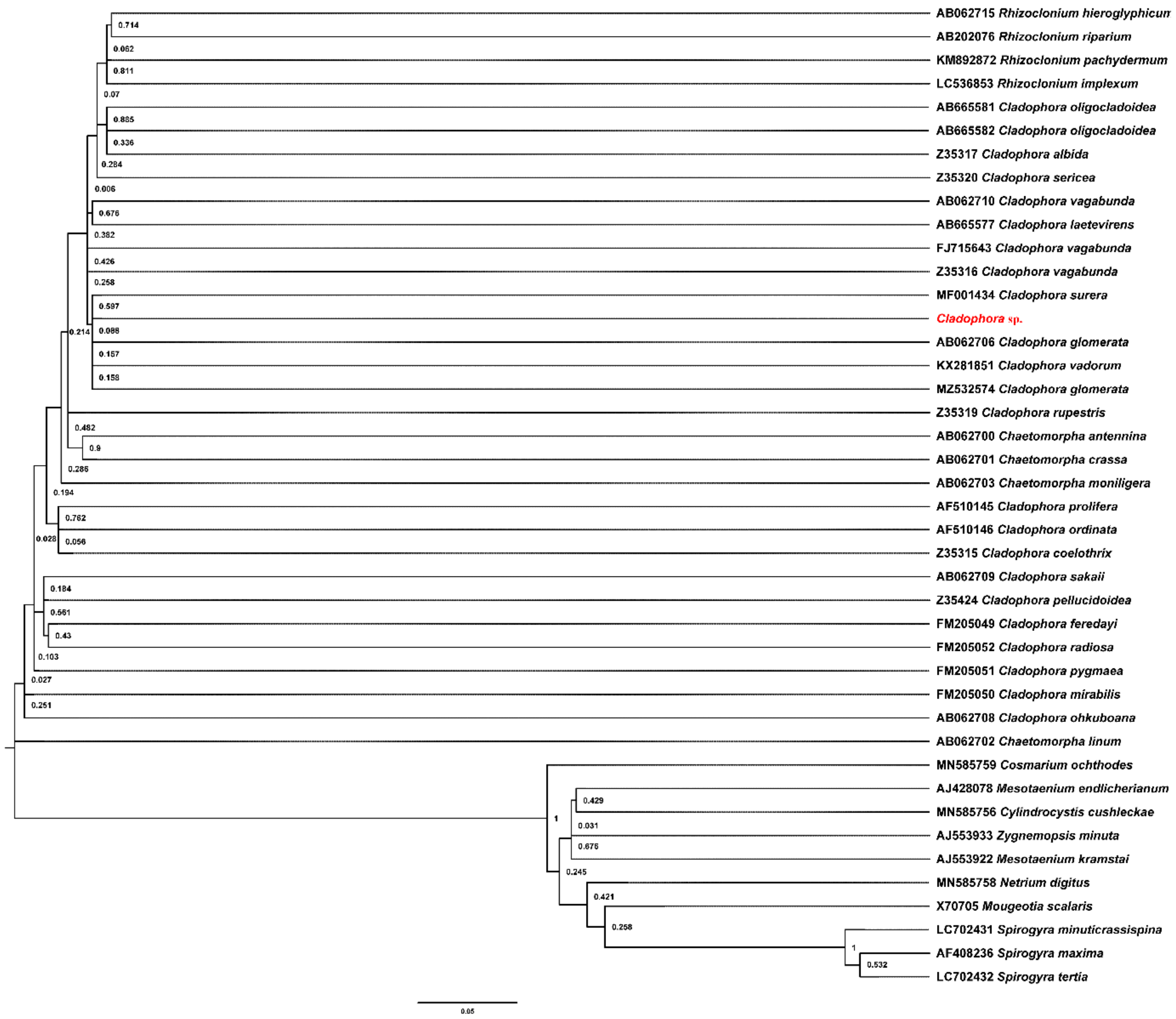

Supplementary Figure S1 Phylogenetic tree of *Cladophora* sp. based on 18S rDNA gene sequences.

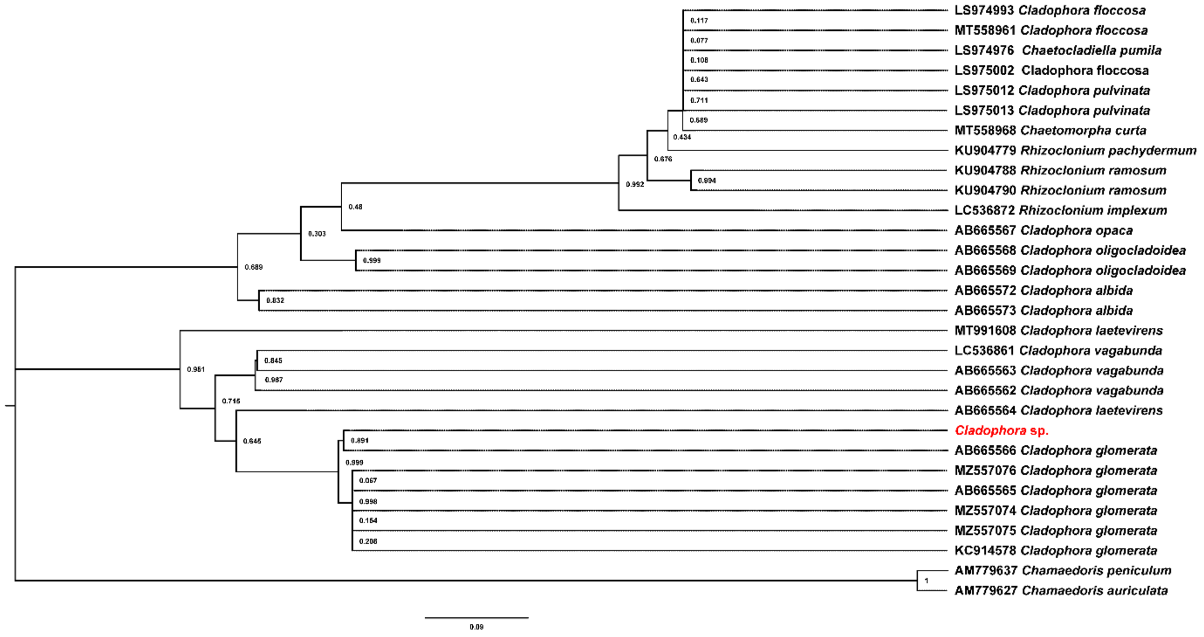

Supplementary Figure S2 Phylogenetic tree of *Cladophora* sp. based on ITS sequences.

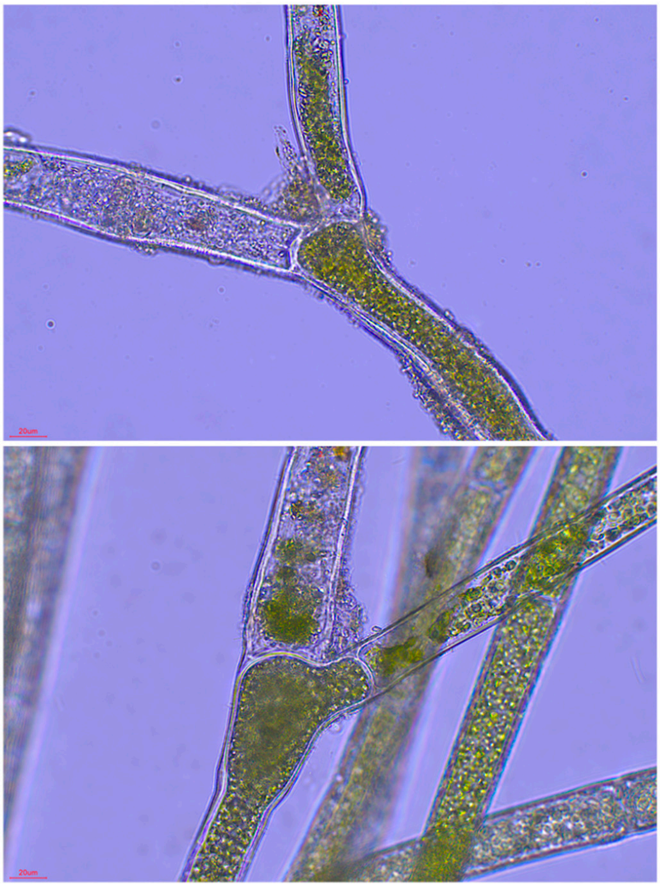

Supplementary Figure S3 The photos of branching conditions of the *Cladophora* sp. used in this study.

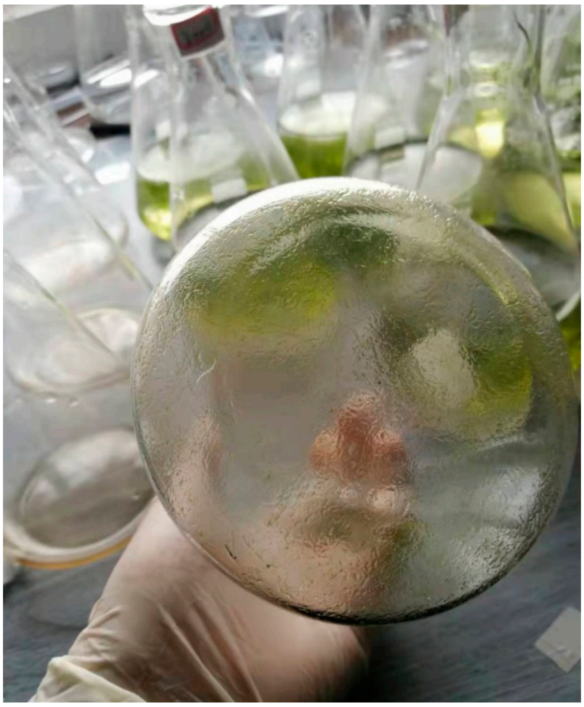

Supplementary Figure S4 The newly germinated seedling of *Cladophora* sp. attached the bottom and side walls of the flasks observed during the growth trials.
